# Genomic Insights into Ciprofloxacin-Resistant Enteropathogenic *Escherichia coli* ST752 in Republic of Korea: A One Health Perspective on Its Emergence and Transmission

**DOI:** 10.3390/antibiotics15030304

**Published:** 2026-03-17

**Authors:** Yeongeun Seo, Wooju Kang, Eunkyung Shin, Jungsun Park, Mooneui Hong, Dong-Hyun Roh, Junyoung Kim

**Affiliations:** 1 Division of Bacterial Diseases, Department of Laboratory Diagnosis and Analysis, Korea Disease Control and Prevention Agency, Cheongju 28159, Republic of Korea; 2 Department of Biological Sciences and Biotechnology, Chungbuk National University, Cheongju 28644, Republic of Korea

**Keywords:** enteropathogenic *Escherichia coli* (EPEC), ciprofloxacin resistance, One Health

## Abstract

**Background/Objectives**: We analyzed the whole-genome sequences of ciprofloxacin-resistant (CIP-R) enteropathogenic *Escherichia coli* (EPEC) ST752 isolates in South Korea to characterize their molecular epidemiology. This lineage has emerged as the predominant CIP-R EPEC clone in South Korea, accounting for 28.8% of human clinical isolates and circulating within the One Health interface. **Methods**: We performed whole-genome sequencing (WGS) and reference-based core-genome single-nucleotide polymorphism (SNP) analysis on 26 CIP-R EPEC ST752 isolates (19 human clinical and 7 poultry-derived isolates). To elucidate their evolutionary history and transmission dynamics, Bayesian phylodynamic and phylogeographic reconstructions were implemented by integrating domestic isolates with a global genome dataset (*n* = 508). **Results**: Isolates from human and poultry sources clustered together with an identical virulence profile and minimal genetic distance. The Bayesian molecular clock analysis estimated that the time to the most recent common ancestor of the South Korean clade was 2000.65. Moreover, the phylogeographic analysis supported statistical evidence (Bayes factor 32.16) for the introduction of this lineage into South Korea from Denmark and revealed a strongly supported host transition from humans to poultry (Bayes factor > 10,000), although this requires cautious interpretation due to limited temporal sampling of poultry isolates. **Conclusions**: Continued integrated One Health surveillance across human, animal, and environmental reservoirs is needed to monitor and prevent the spread of high-risk antimicrobial-resistant clones.

## 1. Introduction

The clinical management of pathogenic *Escherichia coli* infections is difficult because of the global spread of antibiotic resistance (AMR) in *E. coli*, which poses a serious threat to public health [1,2]. In South Korea, genomic surveillance of human clinical isolates has focused primarily on enterohemorrhagic *E. coli* (EHEC) and ESBL-producing strains because of their association with severe foodborne outbreaks [3,4,5,6,7]. Recent national surveillance data (2019–2022) underscore the disproportionate public health impact of enteropathogenic *E. coli* (EPEC). In a large-scale study of 41,227 diarrheal fecal samples, pathogenic *E. coli* was identified as the most frequent bacterial pathogen (27.7%), with EPEC accounting for an overwhelming 85.2% (1647/1934) of these isolates. In contrast, EHEC represented only 5.3% (103/1934) [8]. Given the high clinical prevalence of EPEC and the fact that ciprofloxacin is a primary treatment for acute diarrhea, the increase in the number of ciprofloxacin-resistant (CIP-R) EPEC strains highlights the need for more intensive genomic surveillance systems.

CIP-R *E. coli* is recognized as a major problem not only in humans but also in food, the environment, and animals [9,10,11,12]. Historically, the extensive use of fluoroquinolones in the South Korean poultry industry has increased the resistance rate of *E. coli* isolated from domestic chickens to approximately 70% [13,14]. These poultry environments serve as significant biological reservoirs for the selection of high-risk resistant strains, potentially facilitating their transmission to the human population. However, the specific molecular clones driving the domestic circulation of CIP-R EPEC and their transmission patterns in South Korea’s One Health environment remain poorly understood.

Determining whether the dominant CIP-R EPEC clones in South Korea are a result of recent international introduction or ongoing local evolution is essential for developing effective control strategies. This study aimed to characterize the molecular epidemiology of high-risk CIP-R EPEC strains circulating in South Korea. By integrating domestic isolates with a 25-year global genome dataset and utilizing Bayesian phylodynamic and phylogeographic reconstructions, we aimed to elucidate the evolutionary history, migration routes, and host–ecological niche changes of these clones through human–animal–environment interactions.

## 2. Results

### 2.1. Prevalence and Genomic Relatedness of EPEC ONT:H30 ST752 in South Korea

#### 2.1.1. Distribution in Human and Poultry Populations

An analysis of human clinical isolates revealed that sequence type (ST) 752 is the most prevalent sequence type among CIP-R EPEC, accounting for 28.8% (30/104) of the total isolates. Within this lineage, the ONT:H30 serotype was predominant, and was identified in 63.3% (*n* = 19) of the ST752 cases (Table A1). This identification of the ST752 clone in a clinical setting is consistent with a previous report of this clone in poultry (Table A2) [15]. Therefore, further investigation into the potential epidemiological link between the two reservoirs is warranted.

#### 2.1.2. Phylogenetic Analysis and Genome-Wide SNP Comparisons

**Figure 1:**
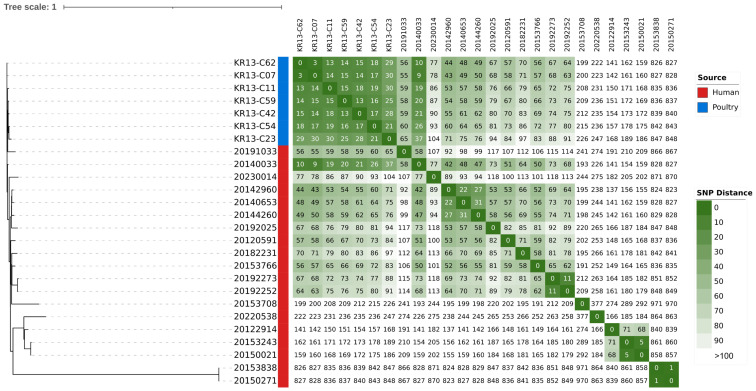
Maximum-likelihood phylogenetic tree and SNP heatmap of ST752 ONT:H30 isolates from human and poultry sources. A phylogenetic tree (**left panel**) was constructed based on core-genome SNPs, with the ciprofloxacin-resistant poultry isolates from 2013 (KR13 series, indicated in blue) clustered closely with human clinical isolates (indicated in red). The heatmap (**right panel**) displays pairwise SNP distances, with the color scale capped at 100 SNPs.

Single-nucleotide polymorphisms (SNPs) were analyzed to evaluate the genetic proximity between human and poultry-derived isolates. The phylogenetic analysis showed that 2013 poultry isolates (KR13-C07 and KR13-C62) were highly related to a 2014 human isolate (20140033), with a genetic distance of only 9–10 SNPs (Figure 1). While the majority of the isolates formed a closely related cluster, two isolates (20150271 and 20153838) exhibited significant divergence, with more than 800 SNPs from the main group.

#### 2.1.3. Quinolone Resistance Mechanisms and Broad-Spectrum Antimicrobial Resistome

The analysis of the resistome of these CIP-R strains revealed several genes and mutations conferring resistance to multiple antimicrobial classes (Supplementary Figure S1). All the isolates harbored point mutations in the quinolone resistance-determining regions (QRDRs) of *gyrA* (S83L, D87N, D87Y, or D87G) and *parC* (S80I). In addition to fluoroquinolones, resistance genes associated with multiple antimicrobial classes were variably detected among the isolates. Several β-lactamase genes, including *bla*_TEM_ variants (*bla*_TEM-1A_, *bla*_TEM-1B_, *bla*_TEM-206_, and *bla*_TEM-214_), which were present in a subset of the isolates, were identified. In addition, the plasmid-mediated AmpC β-lactamase gene *bla*_CMY-2_ was detected in a subset of the isolates.

Genes conferring resistance to aminoglycosides were widely distributed. All the isolates harbored *aph(6)-Id* and *aph(3″)-Ib*, whereas other aminoglycoside resistance genes, such as *aph(3′)-Ia*, *aadA1*, and *aadA2b*, were detected at variable frequencies. Furthermore, resistance genes associated with other antimicrobial classes, including tetracyclines (*tetA* and *tetB*), phenicols (*floR* and *cmlA1*), and sulfonamides (*sul2* and *sul3)*, were sporadically identified. This extensive resistome highlights the ability of the EPEC ONT:H30 ST752 isolates to survive under diverse antimicrobial selective pressures.

#### 2.1.4. Analysis of the Virulence Gene Content and Genotypic Variations

An analysis of virulence factors revealed a highly conserved profile among the EPEC ONT:H30 ST752 isolates, regardless of their host origin (Supplementary Figure S2). All the isolates carried key genes associated with the locus of enterocyte effacement (LEE), including the translocated intimin receptor (*tir*) and type III secretion system effectors such as *espA*, *espB*, and *espF*, although the *tir* gene was notably absent in one isolate. Notably, all the isolates were confirmed to harbor the *eae* gene specifically belonging to the ε (epsilon) subtype. The virulence gene composition of the 2013 poultry isolates (KR13 series) was identical to that of the 2014 human clinical isolate (20140033), demonstrating a high degree of genetic consistency between the two sources. In contrast, isolates 20153838 and 20150271 exhibited expanded virulence profiles, uniquely harboring additional genes such as *iroN*, *iss*, and *tsh*, which were absent in the majority of the other isolates. Despite these variations, the core EPEC-related virulence factors, including the *eae*-ε genotype, remained preserved across the entire ONT:H30 ST752 population.

### 2.2. Phylodynamic and Phylogeographic Analyses of the ST752 Lineage

#### 2.2.1. Temporal Divergence and Evolutionary Dynamics (*n* = 508)

**Figure 2:**
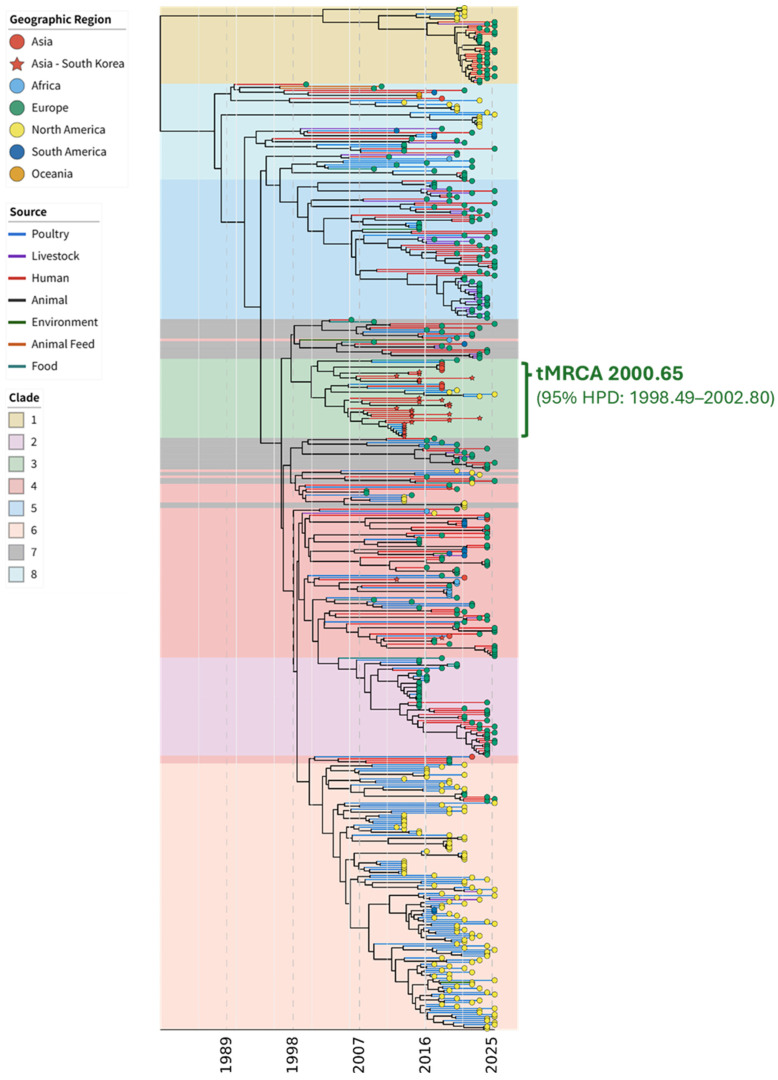
Phylogenetic analysis of global *Escherichia coli* lineages and those from South Korea. Time-scaled maximum clade credibility tree of *E. coli* ST752. Global clades separated using FastBAPS and isolates from South Korea are indicated. The green box shows Global 3 clade sequences from South Korea and the other countries; inferred median MRCA and 95% HPD intervals are indicated for isolates from South Korea. HPD, highest posterior density; MRCA, most recent common ancestor.

The root-to-tip regression analysis of the 508 ST752 isolates revealed a positive temporal signal (*R*^2^ = 0.2688), confirming the suitability of the dataset for molecular clock inference. The time to the most recent common ancestor (tMRCA) of the global ST752 population was estimated to be 1980.6 (95% HPD: 1971.08–1989.50) (Figure 2). For the South Korea clade (within Cluster 3), the tMRCA was inferred at 2000.65 (95% HPD: 1998.49–2002.80), suggesting that this lineage had been circulating domestically for approximately 11 years prior to its initial sampling in 2012. The mean evolutionary rate was estimated at 2.58 × 10^−4^ substitutions per site per year. The Bayesian Skygrid analysis indicated that the ST752 population size remained stable at a low level throughout the 1980s and 1990s. However, a sharp demographic expansion was observed starting around the year 2000, which coincided with the emergence of the South Korean clade (Supplementary Figure S3). This expansion plateaued between 2012 and 2015, with a more than 10-fold increase in the effective population size, which likely reflects the successful global dissemination of CIP-R ST752 clones.

#### 2.2.2. Directional Migration Pathways and Regional Connectivity (*n* = 107)

We selected a representative subset of 107 isolates for the discrete trait analysis to resolve the specific transmission routes of the ST752 lineage while ensuring computational efficiency and mitigating sampling bias from overrepresented regions. This subset was curated to maintain the overall phylogenetic diversity and included isolates from all key geographic locations and host sources. The BSSVS analysis revealed significant directional migration pathways across geographic regions (Table A3). Robust directional migration was identified from Europe to South Korea, supported by a strong Bayes factor (BF = 32.16; posterior probability [PP] = 0.625). While the model identified a specific transition from Denmark, this likely represents a broader European origin for the domestic introduction of the CIP-R ST752 lineage among the sampled locations. Furthermore, South Korea was inferred as a secondary source for further dissemination, with significant outgoing migration toward Japan (BF = 97.98; PP = 0.835) and Lebanon (BF = 71.36; PP = 0.787). Other decisive intercontinental migration events also included transitions from Denmark to South Africa (BF = 364.69) and from the United Kingdom to Belgium (BF = 264.35), highlighting the broad international connectivity of this lineage.

#### 2.2.3. Host-Niche Transition and One Health Transmission Dynamics (*n* = 107)

The asymmetric transition model between isolation sources quantified the directional migration rates among human, animal, and environmental reservoirs (Table A4). The most significant transition was identified from human to poultry sources (BF = 10,160.69; PP = 0.999). Given the temporal concentration of poultry isolates in 2013, this directionality is presented as a strong statistical inference within the current dataset. Conversely, a significant transition from poultry back to humans was also supported (BF = 12.13; PP = 0.594), highlighting the inherent potential for zoonotic transmission within this lineage. Beyond the human—poultry interface, strong evidence was observed for transitions from poultry to swine (BF = 70.88) and from human to animal feed (BF = 31.64). Furthermore, environmental reservoirs appeared to play a crucial role in the transmission cycle, with supported transitions from human to water/river systems (BF = 12.81) and from water/river systems to swine (BF = 11.64), underscoring the environmental persistence and multihost adaptability of the ST752 lineage within a comprehensive One Health framework.

## 3. Discussion

CIP-R EPEC surveillance in South Korea identified ST752 as the predominant lineage among domestic isolates. An analysis of the public genome database further corroborates the global significance of this lineage; ST752 ranks as the third most prevalent sequence type (5.7%) among EPEC isolates worldwide (*n* = 13,488), indicating its status as a major internationally distributed clade. Further characterization of its ecological distribution revealed that in a global dataset of 508 ST752 isolates, poultry-derived isolates comprised the largest proportion (45.8%), supporting the interpretation that poultry represents a major ecological reservoir for this lineage. Previous studies from Europe and the United States similarly reported ST752 as a predominant lineage among poultry-associated isolates, reinforcing the global relevance of poultry-linked maintenance and clonal persistence [16,17]. In South Korea, ST752 has also been reported among chicken-derived *E. coli* isolates from a regional poultry farm survey [15], and a comparative genomic analysis indicated close relatedness between poultry- and human-derived isolates, which is consistent with circulation within a shared domestic interface.

Notably, ONT:H30 ST752 accounted for 28.8% of CIP-R EPEC among human clinical isolates from South Korea. The domestic predominance is likely driven by sustained selective pressure from ciprofloxacin use in both clinical and agricultural settings. Extensive antimicrobial use in the poultry industry, coupled with ciprofloxacin resistance rates approaching 70% among *E. coli* isolates from domestic chickens [13,14], underscores the need for strengthened antimicrobial stewardship to prevent the persistence of resistant clones. The detection of ST752 strains in poultry in 2013, which were within 10 core-genome SNPs from 2014 human clinical isolates, supports that this lineage has established deep endemicity within the South Korean One Health interface. Notably, the composition of virulence factors—specifically the conserved LEE locus and the *eae-ε* genotype—across both human and poultry isolates provides definitive evidence of stable niche sharing.

The distinct serological profile observed in South Korea provides a rationale for using time-resolved phylogenetics to understand its introduction history. While global genomic records demonstrate substantial serological heterogeneity—most notably the widespread prevalence of the H40 flagellar antigen—the domestic population is uniquely characterized by the ONT:H30 profile, suggesting that the South Korean expansion is linked to a specific, internationally circulating yet distinct clade. This marked divergence between the global serological landscape and the predominant Korean profile provides a strong rationale for time-resolved phylogenetic inference to reconstruct the likely introduction history and to define when this clade became established within South Korea. These observations support placing national surveillance isolates from South Korea within a global genomic framework to delineate the population structure and to interpret the distribution of fluoroquinolone resistance and EPEC-associated virulence repertoires within ST752.

Our phylogeographic and host-niche transition models were based on a curated subset of 107 isolates. This approach was essential to minimize potential bias toward countries with higher sampling intensities, such as the UK and US, thereby providing a more balanced reconstruction of global migration and spillover events. While the global common ancestor of ST752 dates to approximately 1980, the South Korean clade emerged in approximately 2000. This result suggests that the lineage circulated domestically for more than a decade before its first clinical detection in 2012. Phylogeographic reconstruction indicates that Europe served as the most likely sampled ancestral region for the introduction of this clone into South Korea. Given that specific countries may act as sampling proxies in global databases, this regional designation more accurately reflects the likely evolutionary origin of the introduction. Following its introduction, South Korea appears to have become a secondary hub, contributing to further dissemination toward Japan and Lebanon.

Our Bayesian phylogeographic reconstruction revealed a complex and highly interconnected transmission network among human, animal, and environmental reservoirs. Interestingly, while poultry is the major reservoir, our phylogeographic model indicates that the transmission directionality is more complex than a simple zoonotic route. A key finding of our study is the directionality of transmission within the host-niche interface. The exceptionally high Bayes factor (BF > 10,000) for the transition from human to poultry sources suggests that human-originated lineages have frequently spilled over into agricultural environments. This transmission pathway is likely mediated by broader systemic flow; human-adapted strains may enter the poultry production chain through environmental bridges such as water systems (BF = 12.81) or contaminated animal feed (BF = 31.64). Specifically, untreated or insufficiently treated wastewater from human settlements and hospitals can serve as a primary vehicle, discharging resistant clones into river systems and irrigation waters [18]. These contaminated water sources, once introduced into the agricultural sector, facilitate the colonization of livestock and poultry. Furthermore, the persistence of these clones in food processing environments, such as slaughterhouses and retail distribution chains, may create additional opportunities for cross-contamination and environmental dissemination. The ability of ST752 to survive across these diverse niches suggests that the environment acts not merely as a passive bridge but as a dynamic reservoir that maintains selective pressure and promotes the circulation of antimicrobial resistance genes among humans and animals [19,20,21]. These findings highlight that human-originated contamination, potentially through wastewater, introduces resistant clones into the agricultural sector, where they encounter further selective pressure from antimicrobial use in livestock [19,20]. However, the lack of longitudinal poultry sampling warrants a conservative interpretation of this specific pathway, and these evolutionary trends should be interpreted as statistical estimates rather than definitive biological conclusions.

Despite these insights, our study has several limitations. The pronounced temporal imbalance between the collection of human isolates (collected over 12 years, 2012–2024) and poultry isolates (limited to 2013) is a notable constraint that may influence the Bayesian transition inference. Specifically, this disparity could lead to the over- or underestimation of host-niche transition rates, particularly regarding events occurring outside the sampled timeframe for poultry. While we integrated a global genomic dataset (*n* = 508) to provide broader evolutionary context, the inference of local transmission directionality remains sensitive to the density and distribution of domestic samples. Furthermore, while subsampling was necessary to mitigate geographic bias, it may have resulted in the loss of low-frequency genetic signals or information regarding rare migration events. Additionally, our discrete trait model assumes that the sampled sources represent the entire ecological diversity of the lineage, which may not fully account for unsampled environmental reservoirs. Therefore, these evolutionary trends and host transitions should be interpreted with caution as statistical estimates rather than definitive biological conclusions.

Future research must implement a more comprehensive and longitudinal One Health surveillance system to overcome these limitations. This system should include extensive environmental sampling of water and soil to better define the role of nonanimal reservoirs in the persistence and circulation of resistant clones. This complex cycle, involving humans, animals, and environmental reservoirs, highlights the urgent need for integrated monitoring to disrupt the transmission pathways of emerging zoonotic pathogens. Overall, our findings support that the South Korean ONT:H30 ST752 lineage represents a locally established and expanded CIP-R EPEC population within a broader, internationally circulating ST752 background. The inferred connectivity across human, poultry, and environmental interfaces highlights that transmission may be mediated through indirect pathways rather than direct host-to-host spread alone. Accordingly, this integrated approach, supported by international guidelines and recent studies, such as coordinated One Health surveillance that integrates clinical, agricultural, and environmental sampling, together with strengthened antimicrobial stewardship, will be essential to limit the persistence and further dissemination of resistant ST752 lineages [12,19,20,22].

In conclusion, we provide insights into the phylogenetic relationships and potential history of the introduction of CIP-R EPEC ST752 into South Korea. Strengthened antimicrobial stewardship and coordinated monitoring are needed to prevent the persistence and dissemination of this lineage within the domestic One Health interface; notably, the role that human-originated environmental contamination can play in the spread of resistant clones should not be underestimated. Continued genomic surveillance across the clinical, agricultural, and environmental sectors remains invaluable for monitoring the evolution of zoonotic pathogens; such efforts could further the design of improved prevention and intervention strategies.

## 4. Materials and Methods

### 4.1. Bacterial Isolates

From 2012 to 2024, a total of 19 CIP-R enteropathogenic *E. coli* strains were isolated from stool or rectal swab samples from patients with diarrhea. These samples were collected and processed according to the standard laboratory diagnosis guidelines of the Korean National Notifiable Disease Surveillance System. Specifically, the isolates were characterized using multiplex PCR targeting the *eaeA*, *bfpA*, *ipaH*, *aggR*, LT/ST, and *stx1*/*stx2* genes. EPEC was defined by the presence of the *eaeA* or *bfpA* genes and the absence of LT/ST and *stx1*/*stx2* genes. Additionally, seven CIP-R *E. coli* strains isolated from chickens in 2013 [15] were included to assess potential zoonotic sources.

### 4.2. Antimicrobial Susceptibility

CIP resistance was tested by broth microdilution using a custom sensitivity panel, KRCDC2F (Trek Diagnostics Systems, Cleveland, OH, USA), and interpreted according to Clinical and Laboratory Standards Institute (CLSI) M100 guidelines (34th edition) [23]. Briefly, the purified bacterial isolates were suspended in distilled water and adjusted to a 0.5 McFarland standard using a nephelometer. Ten microliters of the adjusted suspension was inoculated into 11 mL of cation-adjusted Müller–Hinton (MH) broth and dispensed onto the KRCDC2F Sensititre™ panel using the Sensititre AIM automated dispensing system (Thermo Fisher Scientific, Waltham, MA, USA). The inoculated microplates were sealed with microplate sealing film and incubated in a 37 °C incubator for 18 h. The antimicrobial dispositions of the KRCDC2F panel, which were configured based on the 2014 National Antibiotic Resistance Monitoring System (NARMS) guidelines, are as follows: ciprofloxacin (CIP, 0.03–0.5 μg), nalidixic acid (NAL, 2–128 μg), imipenem (IMI, 1–8 μg), colistin (COL, 2–16 μg), ampicillin (AMP, 2–64 μg), tetracycline (TET, 2–128 μg), chloramphenicol (CHL, 2–32 μg), azithromycin (AZI, 2–32 μg), gentamicin (GEN, 1–64 μg), streptomycin (STR, 2–128 μg;), amikacin (AMI, 4–64 μg), trimethoprim/sulfamethoxazole (SXT, 1/19–16/304), cefotaxime (FOT, 1–32 μg), ceftriaxone (AXO, 1–32 μg), cefoxitin (FOX, 4–32 μg), and ceftazidime (TAZ, 1–16 μg).

### 4.3. Whole-Genome Sequencing

Whole-genome sequencing (WGS) was used for a detailed molecular epidemiological analysis of isolates with fluoroquinolone resistance. Genomic DNA was extracted using the DNeasy Blood and Tissue Kit (Qiagen, Germantown, MD, USA) according to the manufacturer’s instructions. Index-tagged paired-end Illumina sequencing libraries were prepared using NextEra DNA CD Indexes (Illumina, San Diego, CA, USA), and WGS was performed using the Illumina MiSeq platform and the v2 reagent kit (Illumina). The resulting sequence reads were de novo assembled using Qiagen CLC Genomics Workbench software (version 25.0; Aarhus, Denmark), after which adapters and reads < 30 bp in length were removed.

### 4.4. Bioinformatic Analysis

#### 4.4.1. Characteristics of the Isolated Strains

The genomic features of the 26 CIP-R EPEC isolates were characterized using tools from the Center for Genomic Epidemiology (CGE). STs were identified using MLST 2.0, and serotypes (O and H antigens) were determined using SerotypeFinder v2.0. Antimicrobial resistance genes and associated point mutations were detected using ResFinder v4.1, while virulence gene profiles were identified with VirulenceFinder v2.0. Initial molecular epidemiological screening and SNP-based relationships were assessed using CSI Phylogeny v1.4 with *E. coli* K-12 MG1655 (GenBank: NC_000913.3) as the reference, and the results were visualized in iTOL v6.

#### 4.4.2. Dataset Construction and Phylogenetic Reconstruction

Phylodynamic and phylogeographic analyses were performed on the entire ST752 lineage to overcome the limited temporal signal of the ONT:H30 subset (*n* = 23) and provide a broader evolutionary context for its intercontinental dissemination. A total of 765 ST752 genome sequences were retrieved from EnteroBase (as of September 2025). After excluding sequences lacking essential metadata or overlapping with isolates from our study, we selected representative foreign isolates to capture the global genetic diversity. The final dataset included 508 isolates, including 26 isolates from this study and 482 foreign isolates (Supplementary Tables S1 and S2).

Whole-genome SNPs (wgSNPs) were identified using Snippy v4.6.0, mapping all the reads to the *E. coli* K-12 MG1655 reference. Prophage regions identified by PHASTEST [24] and repetitive regions detected via MUMmer v3.23 [25] (--maxmatch --nosimplify) were masked to ensure high-quality alignments. Recombination regions were predicted and removed using Gubbins v3.3.1 [26]. A maximum likelihood (ML) tree was reconstructed from the recombination-free alignment using IQ-TREE v2.2.0 [27] with the GTR+F+ASC+R2 model, as selected by ModelFinder (integrated in IQ-TREE v2.2.0), and 1000 bootstrap replicates. The population structure was further characterized using FastBAPS v1.0.8 [28].

#### 4.4.3. Bayesian Phylogeographic and Phylodynamic Analyses

The temporal signal of the ML tree was assessed using TempEst v1.5.3 [29] through root-to-tip regression (*n* = 508). We identified the optimal priors for Bayesian inference by comparing six model combinations (strict vs. relaxed clocks; Constant, Exponential vs. Bayesian Skygrid tree priors) using stepping-stone (SS) sampling to estimate marginal likelihoods [30]. The best-fit model—comprising a GTR substitution model (with a 4-category Gamma distribution), an uncorrelated relaxed log-normal clock, and a Bayesian Skygrid prior—was selected for the final analysis. We performed representative subsampling to mitigate sampling bias caused by the overrepresentation of closely related isolates from specific countries and periods. Isolates were selected to preserve the overall phylogenetic structure, temporal span, and geographic diversity while near-identical genomes within the same clades were removed. This approach resulted in a refined dataset of 107 representative genomes for downstream phylogeographic and host-niche transition analyses. Spatiotemporal dynamics and the time to the most recent common ancestor (tMRCA) were inferred using BEAST v1.10.5 [31]. An asymmetric discrete trait model with Bayesian stochastic search variable selection (BSSVS) was implemented to identify significant migration routes and host-niche transitions. Five independent MCMC chains were run for 100 million generations each, with sampling every 10,000 steps. Convergence was confirmed in TRACER v1.7.2 (ESS > 200). Runs were combined via LogCombiner v10.5.0 after a 10% burn-in, and the maximum clade credibility (MCC) tree was generated using TreeAnnotator v10.5.0. Finally, the migration route and host transition significance were quantified by calculating the Bayes factor (BF) based on BSSVS outputs. BF values were interpreted according to established criteria, with a BF > 3 considered to indicate statistical significance [32].

## Data Availability

Data available in a publicly accessible repository.

## Supplementary Materials

The following supporting information can be downloaded at: https://www.mdpi.com/article/10.3390/antibiotics15030304/s1, Figure S1: Heatmap of antimicrobial resistance (AMR) gene profiles in ST752 isolates from humans and poultry; Figure S2: Virulence factor (VF) distribution in ST752 isolates from humans and poultry; Figure S3: Bayesian Skyline plot of *Escherichia coli* ST752; Table S1: Metadata and genomic characteristics of the 26 ciprofloxacin-resistant EPEC ST752 study isolates collected in South Korea (2012–2024); Table S2: Summary of metadata for 482 global ST752 reference isolates retrieved from EnteroBase for the comparative genomic analysis.

## Abbreviations

CIP-R	Ciprofloxacin Resistant
ESBL	Extended-Spectrum Beta-Lactamase
EPEC	Enteropathogenic *Escherichia coli*
EHEC	Enterohemorrhagic *Escherichia coli*
ONT	O antigen non-Typeable
AMR	Antimicrobial Resistance
ST	Sequence type
SNP	Single-nucleotide polymorphism
LEE	Locus of Enterocyte Effacement
tir	translocated intimin receptor
QRDR	Quinolone Resistance-Determining Regions
WGS	Whole-Genome Sequencing
MLST	Multi-Locus Sequence Typing
tMRCA	Time to the Most Recent Common Ancestor
HPD	Highest Posterior Density
BSSVS	Bayesian Stochastic Search Variable Selection
BF	Bayes Factor
PP	Posterior Probability
MCMC	Markov Chain Monte Carlo
ESS	Effective Sample Size
MCC	Maximum Clade Credibility
ML	Maximum-likelihood
CLSI	Clinical and Laboratory Standards Institute

## Appendix A

**Table A1:** Metadata of ciprofloxacin-resistant EPEC isolates collected in South Korea for sequence type and serotype distribution analyses (2012–2024).

Sample ID	Year	Source	MLST	Serotype (O:H)
20120101	2012	Human	443	O64:H21
20120346	2012	Human	443	O64:H21
20120537	2012	Human	189	O3:H26
20120591	2012	Human	752	ONT:H30
20120908	2012	Human	10	O70:H40
20121700	2012	Human	382	ONT:H5
20122878	2012	Human	752	O186:H40
20122914	2012	Human	752	ONT:H30
20122958	2012	Human	1193	O75:H11
20122961	2012	Human	189	O80:H26
20130032	2013	Human	189	ONT:H26
20130186	2013	Human	443	O64:H21
20140033	2014	Human	752	ONT:H30
20140653	2014	Human	752	ONT:H30
20142203	2014	Human	752	O25:H48
20142960	2014	Human	752	ONT:H30
20143419	2014	Human	189	ONT:H26
20143760	2014	Human	189	O3:H26
20144260	2014	Human	752	ONT:H30
20144739	2014	Human	189	O80:H26
20145061	2014	Human	443	O64:H21
20145304	2014	Human	382	ONT:H5
20150021	2015	Human	752	ONT:H30
20150271	2015	Human	752	ONT:H30
20153243	2015	Human	752	ONT:H30
20153708	2015	Human	752	ONT:H30
20153766	2015	Human	752	ONT:H30
20153838	2015	Human	752	ONT:H30
20170020	2017	Human	443	O64:H21
20170175	2017	Human	7937	O163:H23
20170186	2017	Human	7937	O169:H23
20170198	2017	Human	443	O64:H21
20170202	2017	Human	7937	O163:H23
20170390	2017	Human	7937	O163:H23
20180732	2018	Human	752	O186:H40
20180866	2018	Human	10	O118:H40
20182231	2018	Human	752	ONT:H30
20183010	2018	Human	642	O61:H4
20190171	2019	Human	443	O64:H21
20190279	2019	Human	7937	O163:H23
20190364	2019	Human	189	O80:H26
20190393	2019	Human	443	O64:H21
20190653	2019	Human	589	O51:H49
20190767	2019	Human	752	O186:H40
20191033	2019	Human	752	ONT:H30
20191192	2019	Human	752	O186:H40
20191830	2019	Human	443	O64:H21
20191881	2019	Human	1193	O75:H5
20192025	2019	Human	752	ONT:H30
20192252	2019	Human	752	ONT:H30
20192273	2019	Human	752	ONT:H30
20192627	2019	Human	443	O64:H21
20192748	2019	Human	382	ONT:H5
20192800	2019	Human	2088	ONT:H49
20193005	2019	Human	7937	O163:H23
20200010	2020	Human	5150	O1:H15
20200031	2020	Human	6272	O138:H48
20200093	2020	Human	1193	O75:H5
20200112	2020	Human	35	O145:H19
20200140	2020	Human	2088	O2:H49
20200191	2020	Human	443	O64:H21
20200227	2020	Human	189	O80:H26
20200253	2020	Human	752	O88:H30
20200255	2020	Human	4119	ONT:H14
20200318	2020	Human	131	O25:H4
20200413	2020	Human	589	O51:H49
20200442	2020	Human	517	O88:H25
20200455	2020	Human	2144	O84:H14
20200536	2020	Human	752	O186:H40
20200993	2020	Human	898	O88:H33
20201569	2020	Human	137	O145:H28
20212789	2021	Human	189	O80:H26
20212809	2021	Human	2144	O84:H5/H14
20213526	2021	Human	517	O88:H25
KR13-C07	2013	Poultry	752	ONT:H30
KR13-C11	2013	Poultry	752	ONT:H30
KR13-C23	2013	Poultry	752	ONT:H30
KR13-C42	2013	Poultry	752	ONT:H30
KR13-C54	2013	Poultry	752	ONT:H30
KR13-C59	2013	Poultry	752	ONT:H30
KR13-C62	2013	Poultry	752	ONT:H30
20220029	2022	Human	189	O80:H26
20220538	2022	Human	752	ONT:H30
20220807	2022	Human	752	O123/O186:H40
20230014	2023	Human	752	ONT:H30
20230024	2023	Human	2569	O124/O164:H4
20230743	2023	Human	189	O3:H26
20230857	2023	Human	744	O101:H9
20230973	2023	Human	18704	ONT:H21
20232022	2023	Human	752	O123/O186:H40
20232036	2023	Human	18704	ONT:H21
20240058	2024	Human	517	O96:H19
20240139	2024	Human	189	O80:H26
20240145	2024	Human	69	O15:H18
20240707	2024	Human	381	O100:H11
20240722	2024	Human	69	O17/O44/O77:H18
20240862	2024	Human	443	O61:H9
20241685	2024	Human	2088	O2:H49
20241697	2024	Human	752	O177:H28
20241863	2024	Human	517	O153:H19
20241874	2024	Human	224	O8:H23
20241893	2024	Human	154	ONT:H38
20241884	2024	Human	4119	ONT:H14
20241895	2024	Human	1033	O156:H1
20242084	2024	Human	18704	ONT:H21

**Table A2:** Metadata of ciprofloxacin-resistant EPEC isolates of ST752 (ONT:H30) collected in South Korea.

Sample ID	Source	Year	Serotype	MLST CC	CIP MIC (µg/mL)	QRDR Mutations		PMQR
						gyrA	parC	
20120591	Human	2012	ONT:H30	ST10 Cplx	8	S83L, D87N	S80I	-
20122914	Human	2012	ONT:H30	ST10 Cplx	8	S83L, D87Y	S80I	-
20140033	Human	2014	ONT:H30	ST10 Cplx	8	S83L, D87N	S80I	-
20140653	Human	2014	ONT:H30	ST10 Cplx	8	S83L, D87N	S80I	-
20142960	Human	2014	ONT:H30	ST10 Cplx	8	S83L, D87N	S80I	-
20144260	Human	2014	ONT:H30	ST10 Cplx	8	S83L, D87N	S80I	-
20150021	Human	2015	ONT:H30	ST10 Cplx	4	S83L, D87Y	S80I	-
20150271	Human	2015	ONT:H30	ST10 Cplx	>16	S83L, D87G	S80I	-
20153243	Human	2015	ONT:H30	ST10 Cplx	2	S83L, D87Y	S80I	-
20153708	Human	2015	ONT:H30	ST10 Cplx	8	S83L, D87N	S80I	-
20153766	Human	2015	ONT:H30	ST10 Cplx	4	S83L, D87N	S80I	-
20153838	Human	2015	ONT:H30	ST10 Cplx	2	S83L, D87G	S80I	-
20182231	Human	2018	ONT:H30	ST10 Cplx	8	S83L, D87N	S80I	-
20191033	Human	2019	ONT:H30	ST10 Cplx	8	S83L, D87N	S80I	-
20192025	Human	2019	ONT:H30	ST10 Cplx	8	S83L, D87N	S80I	-
20192252	Human	2019	ONT:H30	ST10 Cplx	8	S83L, D87N	S80I	-
20192273	Human	2019	ONT:H30	ST10 Cplx	8	S83L, D87N	S80I	-
20220538	Human	2022	ONT:H30	ST10 Cplx	8	S83L, D87G	S80I	-
20230014	Human	2023	ONT:H30	ST10 Cplx	8	S83L, D87N	S80I	-
KR13-C07	Poultry	2013	ONT:H30	ST10 Cplx	8	S83L, D87N	S80I	-
KR13-C11	Poultry	2013	ONT:H30	ST10 Cplx	8	S83L, D87N	S80I	-
KR13-C23	Poultry	2013	ONT:H30	ST10 Cplx	8	S83L, D87N	S80I	-
KR13-C42	Poultry	2013	ONT:H30	ST10 Cplx	16	S83L, D87N	S80I	-
KR13-C54	Poultry	2013	ONT:H30	ST10 Cplx	8	S83L, D87N	S80I	-
KR13-C59	Poultry	2013	ONT:H30	ST10 Cplx	16	S83L, D87N	S80I	-
KR13-C62	Poultry	2013	ONT:H30	ST10 Cplx	8	S83L, D87N	S80I	-

## Appendix B

**Table A3:** Results of Bayesian phylogeographic analysis of *E. coli* ST752 clades: Geographic migration among different countries.

Transition	Posterior Probability	Bayes Factor
Denmark → South Africa	0.9497	364.69
United Kingdom → Belgium	0.932	264.35
Denmark → Ecuador	0.8793	140.59
South Korea → Japan	0.8355	97.98
Denmark → Germany	0.8063	80.31
South Korea → Lebanon	0.7871	71.36
Denmark → Canada	0.6602	37.49
Denmark → South Korea	0.625	32.16
Canada → United States	0.6226	31.83
Denmark → Brazil	0.6071	29.82
Denmark → United States	0.6055	29.62
South Africa → Indonesia	0.4808	17.87
Brazil → Mexico	0.4805	17.85
Denmark → United Kingdom	0.4407	15.21
Brazil → Australia	0.432	14.67
Denmark → Uganda	0.4145	13.66
Denmark → Netherlands	0.3712	11.39
United States → Canada	0.3646	11.07
United States → United Kingdom	0.3638	11.03
Germany → United Kingdom	0.3512	10.45
Denmark → Bangladesh	0.3339	9.67
United States → China	0.3269	9.37
Denmark → Spain	0.3028	8.38
United Kingdom → Netherlands	0.2911	7.92
Canada → United Kingdom	0.2845	7.67
Denmark → Indonesia	0.2607	6.81
Denmark → Norway	0.2429	6.19
Denmark → China	0.2322	5.84
Netherlands → United Kingdom	0.2276	5.68
Japan → Lebanon	0.1974	4.75
Denmark → Australia	0.1924	4.6
Brazil → Spain	0.1906	4.55
Spain → Norway	0.1896	4.51
Canada → China	0.1854	4.39
Lebanon → Japan	0.1753	4.1
Brazil → Norway	0.172	4.01
Germany → Netherlands	0.1709	3.98
Norway → Spain	0.1701	3.96
Denmark → Mexico	0.1699	3.95
Ecuador → Uganda	0.1476	3.34
Mexico → Brazil	0.1446	3.26
Germany → Uganda	0.1393	3.12

**Table A4:** Results of Bayesian phylogeographic analysis of *E. coli* ST752 clades: Transitions between host and environmental sources.

Transition	Posterior Probability	Bayes Factor
Human → Poultry	0.9992	10,160.69
Poultry → Swine	0.8953	70.88
Human → Animal_Feed	0.7925	31.64
Poultry → Invertebrate	0.7674	27.33
Human → Water/River	0.6073	12.81
Poultry → Human	0.5942	12.13
Water/River → Swine	0.5842	11.64
Poultry → Bovine	0.4443	6.62
Human → Animal-related	0.3702	4.87
Poultry → Water/River	0.3661	4.78
Poultry → Canine	0.3591	4.64
Human → Canine	0.3499	4.46
Human → Environment	0.3184	3.87
Swine → Water/River	0.3058	3.65
Poultry → Animal-related	0.2844	3.29
Poultry → Environment	0.2835	3.28
