# Supplementary material for: Genomic Insights into Ciprofloxacin-Resistant Enteropathogenic Escherichia coli ST752 in Republic of Korea: A One Health Perspective on Its Emergence and Transmission
Source: Antibiotics (Basel). 2026 Mar 17;15(3):304. doi: 10.3390/antibiotics15030304 (PMC13024096; doi:10.3390/antibiotics15030304)
Supplement: Supplementary file 1 [file antibiotics-15-00304-s001.zip › antibiotics-4168379-Supplementary Materials.pdf]

Supplementary Materials

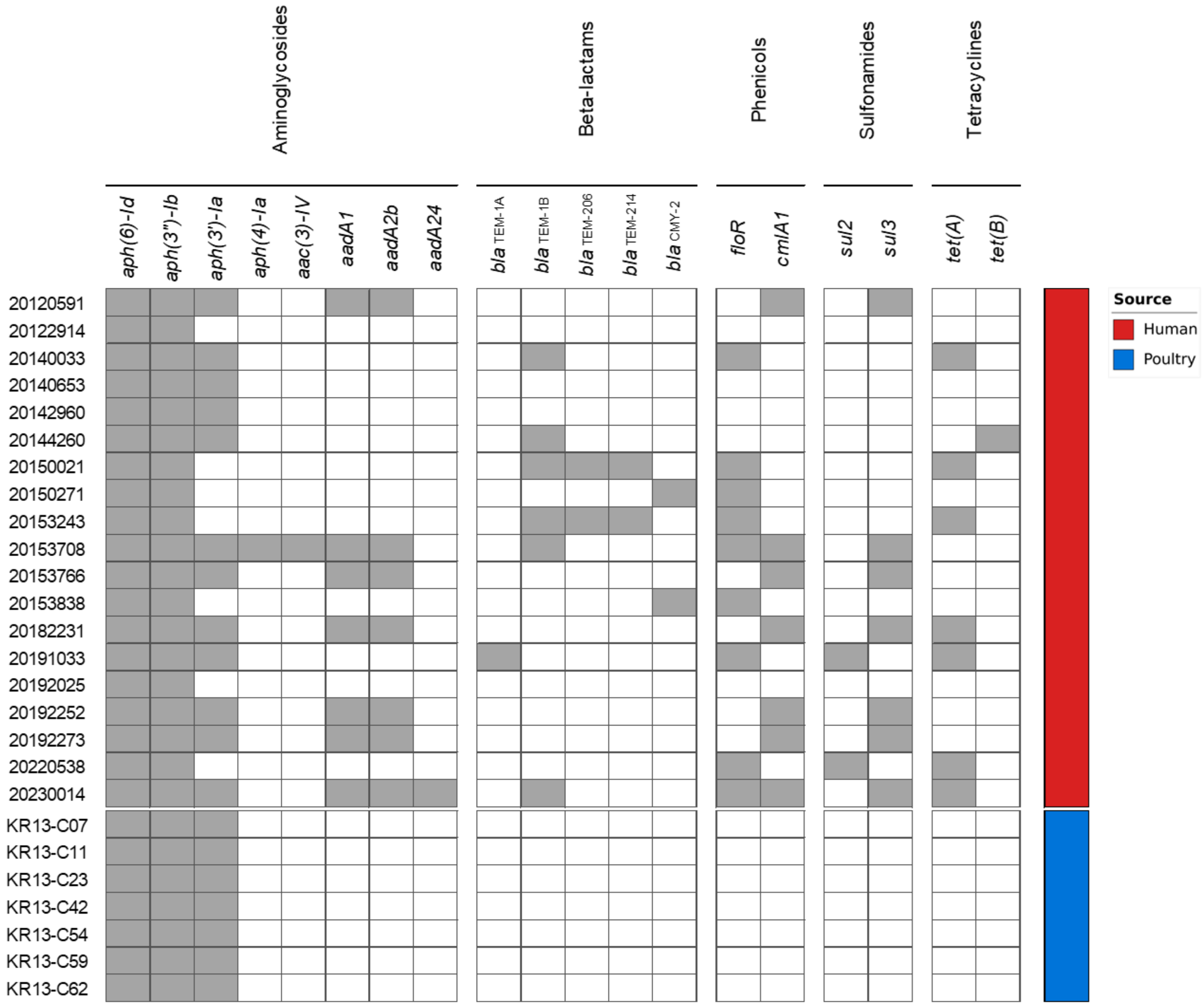

Supplementary Figure S1. Heatmap of antimicrobial resistance (AMR) gene profiles in ST752 isolates from humans and poultry.

The heatmap illustrates the presence (gray) or absence (white) of diverse AMR determinants categorized by their respective antibiotic classes: aminoglycosides, beta-lactams, phenicols, sulfonamides, and tetracyclines. The isolates are color-coded by source on the right bar, with human clinical isolates indicated in red and poultry-derived isolates (KR13 series) indicated in blue.

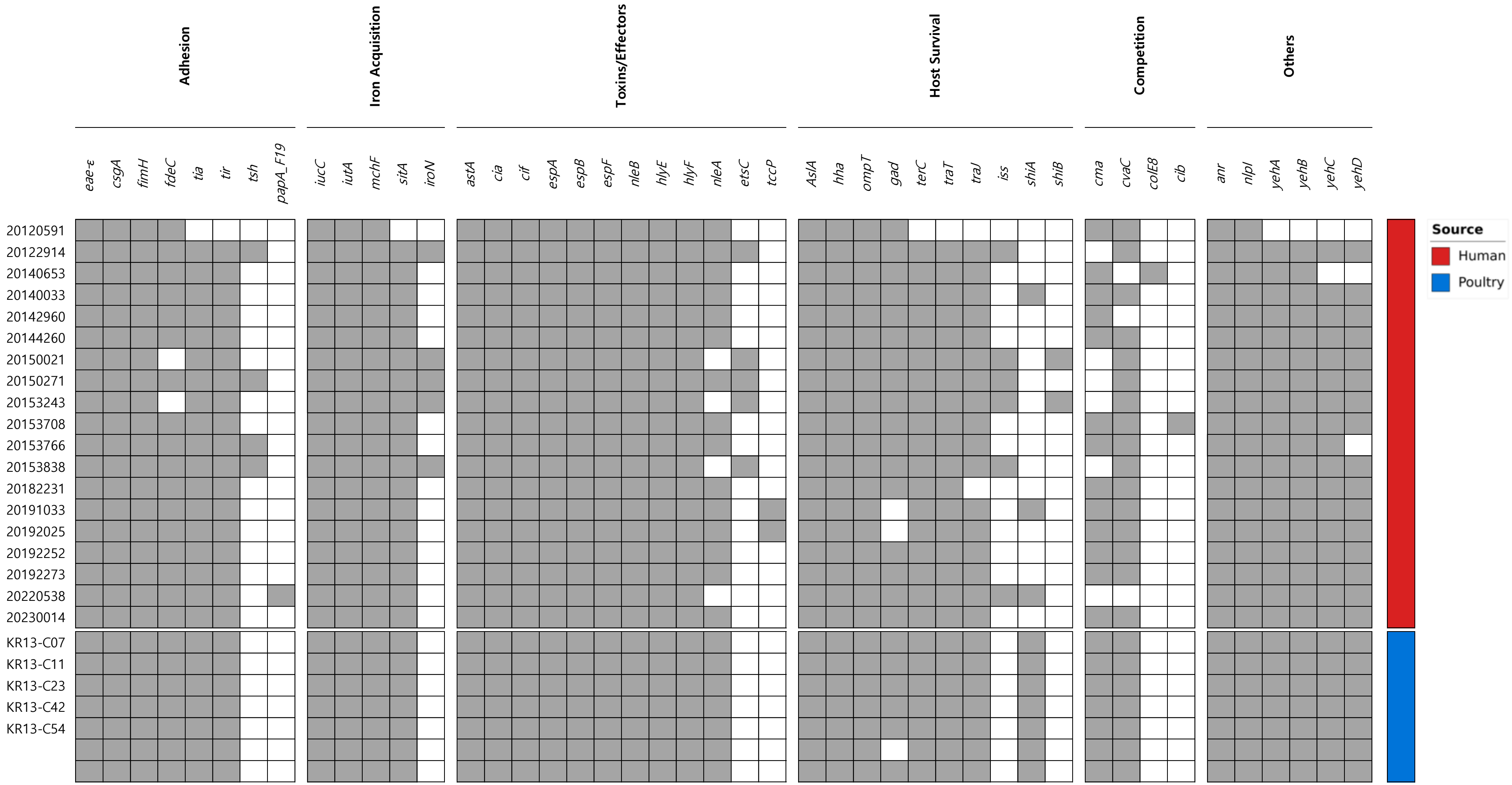

Supplementary Figure S2. Virulence factor (VF) distribution in ST752 isolates from humans and poultry.
The heatmap illustrates the presence (gray) and absence (white) of virulence determinants across six functional categories: adhesion, iron acquisition, toxins/effectors, host survival, competition, and others. Isolates are categorized by source at the bottom, with human clinical isolates indicated in red and poultry-derived isolates (KR13 series) in blue.

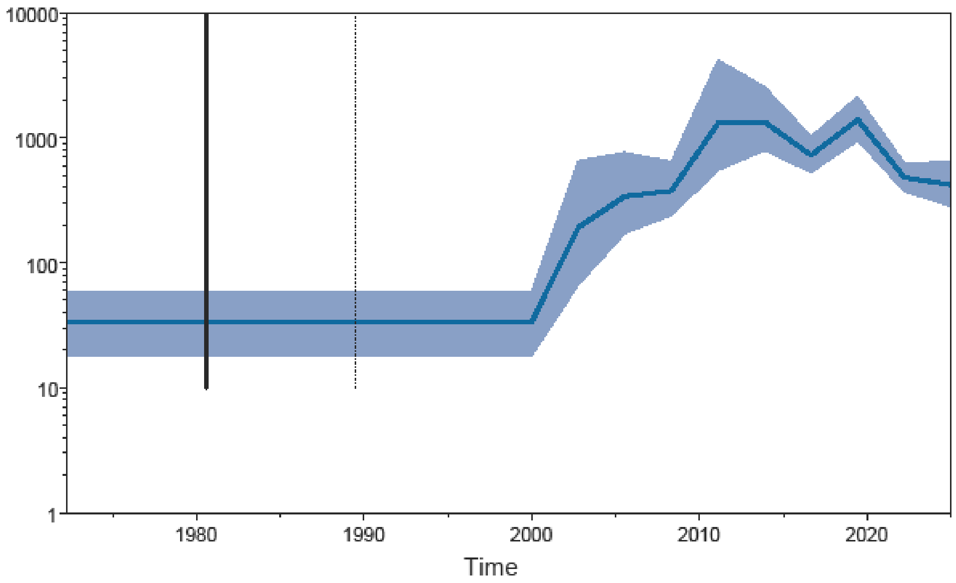

Supplementary Figure S3. Bayesian Skyline plot of *Escherichia coli* ST752.
The x-axis denotes the time in years, and the y-axis represents the effective population size on a logarithmic scale. The solid blue line indicates the median estimate of the population size, with the blue shaded region representing the 95% highest posterior density (HPD) interval. The solid vertical line indicates the median tMRCA (1980.6; 95% HPD, 1971.08–1989.50), which represents the estimated time of the most recent common ancestor for this lineage.

Supplementary Table S1. Metadata and genomic characteristics of the 26 ciprofloxacin-resistant EPEC ST752 study isolates collected in South Korea (2012–2024).

Supplementary Table S2. Summary of metadata for 482 global ST752 reference isolates retrieved from EnteroBase for the comparative genomic analysis.
